# The pan-HDAC inhibitor panobinostat acts as a sensitizer for erlotinib activity in *EGFR*-mutated and -wildtype non-small cell lung cancer cells

**DOI:** 10.1186/s12885-015-1967-5

**Published:** 2015-12-16

**Authors:** Gabriele Greve, Insa Schiffmann, Dietmar Pfeifer, Milena Pantic, Julia Schüler, Michael Lübbert

**Affiliations:** 1 University of Freiburg Medical Center, Freiburg, Germany; 2 University of Freiburg, Faculty of Biology, Freiburg, Germany; 3 University of Freiburg, Faculty of Medicine, Freiburg, Germany; 4 Department for in vivo Tumorbiology, Oncotest GmbH, Freiburg, Germany; 5 DKTK, German Consortium for Translational Cancer Research, Freiburg, Germany

**Keywords:** Epigenetic therapy, Non-small cell lung cancer, Erlotinib, Chromatin modification, Histone acetylation, Histone methylation, TKI

## Abstract

**Background:**

The receptor tyrosine kinase (RTK) EGFR is overexpressed and mutated in NSCLC. These mutations can be targeted by RTK inhibitors (TKIs) such as erlotinib. Chromatin-modifying agents may offer a novel therapeutic approach by sensitizing tumor cells to TKIs.

**Methods:**

The NSCLC cell lines HCC827 (*EGFR* mutant, adenocarcinoma), A549 (*EGFR* wt, adenocarcinoma) and NCI-H460 (*EGFR* wt, large cell carcinoma) were analyzed by SNP6.0 array. Changes in proliferation after panobinostat (LBH-589, PS) and erlotinib treatment were quantified by WST-1 assay and apoptosis by Annexin V/7-AAD flow cytometry. Abundance of target proteins and histone marks (acH3, H3K4me1/2/3) was determined by immunoblotting.

**Results:**

As expected, the *EGFR* wt cell lines A549 and NCI-H460 were quite insensitive to the growth-inhibitory effect of erlotinib (IC_50_ 70-100 μM), compared to HCC827 (IC_50_ < 0.02 μM). All three cell lines were sensitive to PS treatment (IC_50_: HCC827 10 nM, A549 20 nM and NCI-H460 35 nM). The combination of both drugs further reduced proliferation in HCC827 and in A549, but not in NCI-H460. PS alone induced differentiation and expression of p21^WAF1/CIP1^ and p53 and decreased CHK1 in all three cell lines, with almost no further effect when combined with erlotinib. In contrast, combination treatment additively decreased pEGFR, pERK and pAKT in A549. Both drugs synergistically induced acH3 in the adenocarcinoma lines. Surprisingly, we also observed induction of H3K4 methylation marks after erlotinib treatment in HCC827 and in A549 that was further enhanced by combination with PS.

**Conclusion:**

PS sensitized lung adenocarcinoma cells to the antiproliferative effects of erlotinib. In these cell lines, the drug combination also had a robust, not previously described effect on histone H3 acetylation and H3K4 methylation.

## Background

Lung cancer remains the leading cause of cancer-related death worldwide [1]. For stage IIIB/IV non-small cell lung cancer (NSCLC) patients, the median survival with standard chemotherapy is approximately 10 months [2]. While treatment with tyrosine kinase inhibitors (TKIs) can dramatically prolong survival in a subgroup of patients, their therapeutic index depends heavily on the patients’ Epidermal Growth Factor Receptor (*EGFR*) mutational status [3]. While reversible first-generation TKIs like erlotinib and gefitinib are superior to chemotherapy in *EGFR*-mutated patients, they show less activity in patients with wildtype (wt) *EGFR* [4, 5]. Different attempts have been undertaken to sensitize *EGFR* wt NSCLC cells to the antineoplastic effects of TKIs, including combination therapy with epigenetically active drugs [6].

Histone deacetylase inhibitors (HDACis) exert their anticancer effects by increasing acetylation of core histones as well as non-histone proteins, thereby influencing gene transcription and ultimately leading to the induction of apoptosis, differentiation or degradation of misfolded proteins [7]. To date, three HDACis, vorinostat (SAHA), romidepsin and panobinostat (LBH-589, PS), have been FDA-approved for the treatment of cutaneous and peripheral T-cell lymphoma and multiple myeloma. In contrast, in solid tumors single-agent treatment with HDACis exhibits only a limited clinical benefit [8]. However, the combination with different cancer therapeutics (chemo-, radio- or antihormonal therapy as well as TKIs), has demonstrated increased efficacy in several settings [9, 10].

HDACis and TKIs affect common downstream pathways: both alter the expression of cell cycle regulators such as p21^WAF1/CIP1^, p53 or CHK1 [11, 12]. Downregulation of CHK1 was recently shown to be a potential pharmacodynamic biomarker for HDACi response in NSCLC patients and was negatively correlated with the expression of E-cadherin [13]. On the other hand, E-Cadherin is also an important protein of interest, as its loss leads to both metastatic spread and resistance to TKI treatment, and it was already shown to be upregulated by HDACi *in vitro* [14]. Thus, high E-Cadherin expression is not only a prognostic marker for a better outcome in lung cancer but also correlates with response to TKIs [14]. Non-toxic treatment regimens combining HDACis and TKIs have been established [13, 15], and first clinical trials showed that the combination therapy is especially effective in patients expressing high E-Cadherin levels [16].

Recent publications could also demonstrate a crosstalk between HDACi induced histone acetylation and histone methylation that resulted in differentiation and induction of tumor suppressor genes, such as p21^WAF1/CIP1^ [17, 18].

Here, we utilized three NSCLC cell lines with different genotypes (regarding *EGFR* and *KRAS* mutational status, different copy number gains and losses of genes relevant for lung cancer) to investigate the molecular mechanisms of the combination of the pan-HDACi PS with erlotinib. In this study, we could show that the combination of erlotinib with PS indeed exerts additive antineoplastic effects upon the *EGFR*-mutated adenocarcinoma cell line HCC827 and the *EGFR* wt adenocarcinoma cell line A549, whereas this effect was not seen in the large-cell carcinoma cell line NCI-H460. Interestingly, the deposition of activating histone marks mediated by HDACi treatment with PS, including histone methylation marks, was enhanced by erlotinib, an effect of the TKI that has not been described previously.

## Methods

### Cell lines

Three non-small cell lung cancer lines HCC827, A549 and NCI-H460 (ATCC, American Type culture collection, Manassas, VA; USA; DSMZ, Braunschweig, Germany) with different histological properties as well as EGFR and KRAS mutational status were cultured in RMPI 1640 (Gibco, Thermo Fisher Scientific Inc.) incl. 10 % FCS (Biochrome, Merck Millipore, Berlin, Germany) and 100U/ml penicillin/streptomycin (Gibco) at 37 °C and 5 % CO_2._

HCC827 is an adenocarcinoma line with the E746 - A750 *EGFR* deletion [19]. A549 is of the same histological NSCLC subtype and harbors a *KRAS* mutation, but is EGFR wildtype [20]. NCI-H460 is a large-cell carcinoma line, also with mutated *KRAS* and wildtype *EGFR* [21]. As published in the COSMIC database, none of the three cell line harbors a *TP53* mutation (http://cancer.sanger.ac.uk/ cancergenome/projects/cosmic/).

Since we only used commercially available cell lines, no approval of an appropriate ethics committee was needed.

### Treatment of cells, growth inhibition and measurement of apoptosis

IC_50_ concentrations of panobinostat (LBH-589, PS, LC laboratories, Woburn, MA, USA) and erlotinib (LC laboratories) were determined by WST-1 assay (Roche Applied Science, Mannheim, Germany). Briefly, 5x10^2^/ml cells were seeded in 96-well plates, treated on three consecutive days with different concentrations of PS or erlotinib and changes in proliferation were measured according to manufacturer’s protocol. IC_50_ values were reached when cell growth was inhibited to 50 % of the DMSO control. As previously described, the IC_50_ concentrations for PS were 10nM for HCC827, 20 nM for A549 and 35 nM for NCI-H460 [22]. Since the EGFR wt cells were highly insensitive to erlotinib a dose of 2 μM was used. For treatment of EGFR mutated HCC827 cells a non-toxic concentration of 10 nM erlotinib was used.

Both PS and erlotinib were dissolved in DMSO (Sigma) in stock solution of 1 mM and 10 mM, respectively, and diluted to the corresponding concentrations directly prior to the treatment for each timepoint.

Cell death was observed by trypan blue exclusion assay and by flow cytometry measurement with Annexin V (eBioscience, Frankfurt a.M., Germany) and 7-AAD (BD Pharmingen, Heidelberg, Germany) staining.

### Western blot

After treatment with the corresponding IC_50_-dose of PS and 2 μM (10 nM, respectively) erlotinib as single agents and in combination, 5x10^6^ NSCLC cells per condition were harvested after 24, 48 and 72 h. DMSO as well as medium-only served as negative controls. Whole cell lysis and protein isolation was performed according to protocol (Active Motif) and protein concentration was measured by BCA assay (Thermo Scientific). Equal amounts of protein were separated using 4-12 % gradient Bis/Tris acrylamide gels in the NuPAGE electrophoresis and blotting system (Invitrogen, Thermo Scientific).

Antibodies against phospho- and total ERK, phospho- and total AKT, phospho-and total CHK1, β-catenin (Cell Signaling Technology), E-cadherin (BD Transduction Laboratories), acetylated histone H3, H3K4me1,–2,–3 (Merck Millipore), p21^WAF1/CIP1^, p53, GAPDH, alpha-tubulin, and HRP-labeled secondary antibodies against goat, rabbit and mouse (Santa Cruz Biotechnology) were used. ECL Plus Western Blotting Detection System (Thermo Scientific) served as substrate for chemoluminescence.

### SNP-array analysis

DNA was isolated from 5x10^6^ cells per cell line according to manual with the Blood & Tissue DNeasy Mini Kit (Qiagen). DNA concentration was measured with NanoDrop1000 (Thermo Scientific).

500 ng of genomic DNA were used for SNP array analysis (Genome-Wide Human SNP Array 6.0 Affymetrix). Copy number variations were analyzed with the Genotyping Console Software (Affymetrix).

[GEO:GSE63784] (http://www.ncbi.nlm.nih.gov/geo/query/acc.cgi?acc=GSE63784).

### Statistics

Statistical analysis of data was performed using the GraphPad Prism 5.0 software with Student’s *t*-test to test differences between two treatment groups and a linear regression model for the identification of the corresponding IC_50_ values.

## Results

### Characterization of three NSCLC cell lines reveals copy number variations in oncogenes and tumor suppressor genes

Before treatment of the cell lines HCC827, A549 and NCI-H460, we wished to confirm and extend the genetic information given by the provider. We analyzed copy number (CN) variations by SNP array, since copy number amplifications of *EGFR*, *MYC* and *KRAS* and *TP63* are often described in NSCLC. Specifically *EGFR* mutated cells like HCC827 display an amplification of the oncogene [23] which we could confirm (Table 1). Similarly, amplification of *MYC* was validated for the NCI-H460 cells (with a concomitant CN reduction of *EGFR).* Amplifications of *KRAS* could be confirmed for A549 (KRAS mut) and the *KRAS* wt HCC287 [24]. None of the three cell lines showed amplifications of *MET,* which are closely linked to TKI resistance [25, 26]. No CN gains or losses were detected for *ALK, BRAF* and *PI3K*, (Table 1).

**Table 1:** Copy number variations of oncogenes and tumor suppressors in three NSCLC cell lines

gene	ALK	BRAF	CDKN1A	EGFR	KRAS	MET	MYC	TP53	TP63	PI3K
HCC827	n	n	n	4	3	n	4	3	n	n
A549	n	n	1	3	3	n	3	n	1	n
NCI-H460	n	n	1	1	n	n	4	n	n	n

Copy numbers of selected genes in HCC827, A549 and NCI-H460 were determined by Affymetrix SNP Array 6.0

0 = complete loss, 1 = 50 % loss, n = no loss or gain, 3 = 50 % gain and 4 = 100 % gain of genomic material, relative to ploidy (all percentages are approximate values computed with Affymetrix Genotyping Console Software)

### Panobinostat enhances the antiproliferative effect of erlotinib in *EGFR*-mutant and -wildtype cell lines

**Fig. 1:**
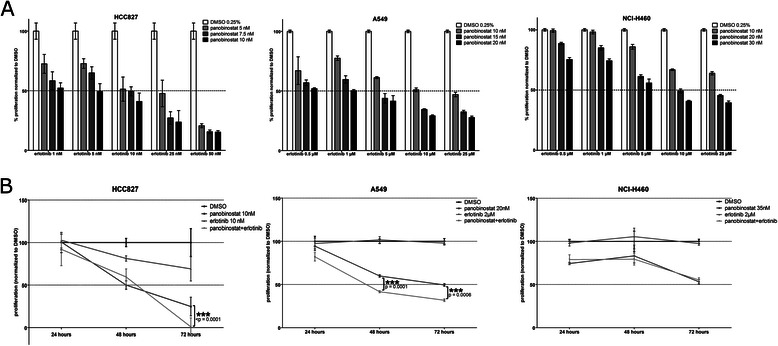
**a** Combination dose-finding studies with panobinostat and erlotinib. The three NSCLC cell lines HCC827, A549 and NCI-H460 were treated with three different concentrations of PS in combination with five concentrations of erlotinib to determine by WST-1 assay the most effective combination dose that reduces proliferation to 50 % compared to the vehicle (DMSO) control after 72 h (representative experiment with biological triplicates shown). The concentration ranges of both drugs were determined beforehand in independent experiments (data not shown). **b** Combination treatment with panobinostat and erlotinib significantly reduces proliferation of NSCLC adenocarcinoma cell lines. Proliferation of NSCLC cells after treatment with PS and/or erlotinib was measured by WST-1 assay after 24, 48 and 72 h, normalized to DMSO vehicle treated cells. The combination of PS and erlotinib reduced proliferation significantly after 72 h in HCC827 and A549 cells (compared to PS alone; *** *p* < 0.001 Student’s *t*-test, paired, two-tailed)

We next examined growth-inhibitory effects of erlotinib and PS, alone and in combination. NSCLC patients usually receive a daily dose of 150 mg erlotinib, which results in plasma levels between 1 and 3 μM [27]. Thus we chose to treat the *EGFR* wildtype cells with an erlotinib concentration of 2 μM. Additionally, we performed extensive dose-findings to determine the most effective but also least toxic doses for PS in combination with erlotinib (Fig. 1a).

**Fig. 2:**
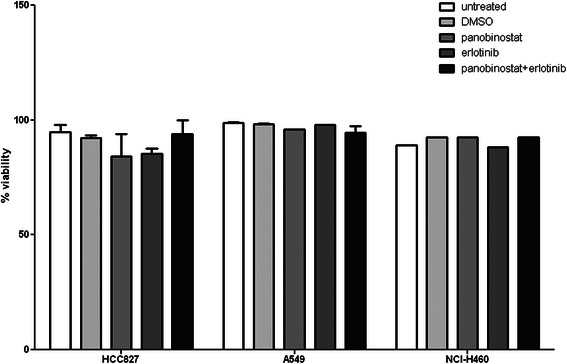
Combination treatment with panobinostat and erlotinib shows no increase in cytotoxicity after 72 h. Viability of NSCLC cells after 72 h of treatment with PS and/or erlotinib measured via Trypan blue exclusion assay. No significant induction of cell death was measured. This was also validated by flow cytometry with Annexin V and 7-AAD (data not shown)

As expected, proliferation of A549 and NCl-H460 cells was not inhibited by erlotinib alone, while *EGFR* mutant HCC827 cells showed striking growth inhibition already at 10 nM (Fig. 1b). Treatment with PS at nanomolar doses impaired proliferation in all three cell lines, with HCC827 being most sensitive [22]. When combining PS and erlotinib, a time- and dose-dependent growth inhibition could be observed, with a significantly additive effect of PS across the entire dose range in HCC827 and A549. In NCI-H460, erlotinib showed no additional effect. (Fig. 1b). Inhibition of proliferation was not accompanied by an increase in cytotoxicity and apoptosis (Fig. 2).

### Combined panobinostat/erlotinib treatment reduces phospho-EGFR

Binding of its ligands leads to EGFR dimerization and autophosphorylation, allowing the recruitment of its downstream targets. Therefore, phosphorylation is an indicator for activated EGFR.

**Fig. 3:**
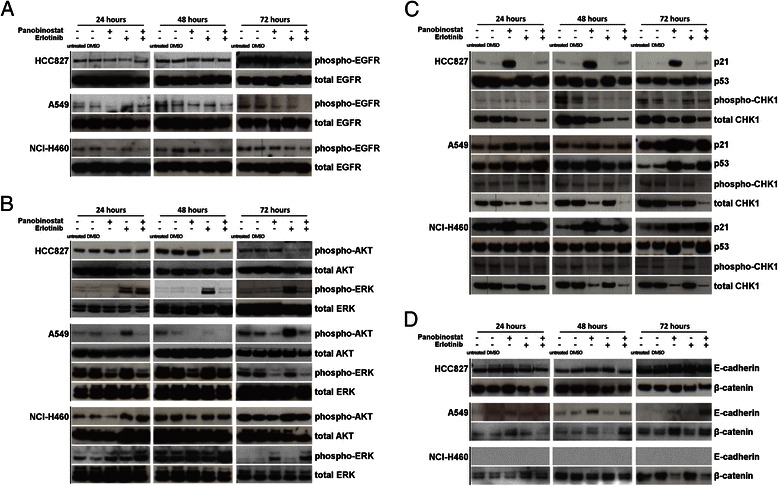
**a** Panobinostat and erlotinib cooperate in reducing the abundance of phospho-EGFR in NSCLC cell lines. Western Blot analysis of the TKI-sensitive cell line HCC827 and the TKI-insensitive cell lines A549 and NCI-H460 was performed using antibodies against the phosphorylated EGF-receptor and, serving as control, total EGFR. **b** Panobinostat and erlotinib differentially regulate phospho-AKT and phospho-ERK. Western blot analysis of the proliferation and survival promoting proteins of the MAPK-pathway: phospho-ERK (compared to total ERK), and of the PI3K-pathway: phospho-AKT (compared to total AKT). **c** Regulation of cell cycle proteins p21^WAF1/CIP1^, p53 and CHK1 by panobinostat and erlotinib combination treatment. Western blot analysis after 24, 48 and 72 h of treatment with PS and/or erlotinib was performed using antibodies against p21^WAF1/CIP1^, p53, phosphorylated CHK1 (Ser280) and total CHK1. An induction of p21^WAF1/CIP1^ and p53 after PS as well as combination treatment was detectable in both EGFR *wt* cell lines A549 and NCI-H460 only. A downregulation of CHK1 after PS and combination treatment was seen in all three cell lines. **d** Panobinostat alone and in combination with erlotinib upregulates E-Cadherin and β-catenin in the EGFR wildtype NSCLC adenocarcinoma cell line A549. Western blot analysis of the three NSCLC lines after 24, 48 and 72 h of combination treatment was performed using antibodies against E-Cadherin and β-Catenin

As shown in Fig. 3a, after 72 h PS slightly reduced phospho-EGFR in both *EGFR* wt cell lines whereas no effect could be detected in HCC827. As expected, a response to erlotinib could be seen in HCC827, but also A549 showed a decrease in phospho-EGFR. Combining the two compounds further downregulated phospho-EGFR in HCC827, A549 and, to a lesser extent, also in NCI-H460.

### Panobinostat and erlotinib affect the expression of downstream targets of the EGFR pathway

EGFR is located upstream of a signaling cascade that includes the PI3K/AKT- and RAS/MAPK-pathways and regulates cell differentiation and proliferation. Western Blot was performed to investigate the effects of erlotinib ± PS on the expression and phosphorylation of EGFR downstream proteins AKT and ERK.

In HCC827, erlotinib diminished phospho-AKT almost completely; this effect could not be further increased by PS. Unexpectedly, single agent erlotinib was not only unable to decrease the already low, phospho-ERK levels, but rather induced phosphorylation of ERK. In A549, erlotinib had no effect on phospho-ERK but induced the expression of phospho-AKT, whereas PS reduced phosphorylation of AKT and ERK. In NCI-H460, erlotinib had no effect on phospho-AKT or phospho-ERK. PS alone could only slightly reduce the phosphorylation of AKT, but, in contrast, increased phosphorylation of ERK after 72 h. As in A549, no cooperative effect of the combination could be detected (Fig. 3b).

### In *EGFR* mutated cells, erlotinib counteracts panobinostat-induced increase of p21 ^WAF1/CIP1^ and p53 and decrease of CHK1

The p53 pathway reacts to different stress signals caused by e.g. DNA damage or hypoxia. Its downstream targets include the CDK inhibitor p21^WAF1/CIP1^, which however can also be induced p53-independently [28]. Both cooperatively downregulate CHK1 [29]. Additionally, AKT further inhibits CHK1 function by phosphorylation of Ser280. As already shown, HDAC inhibition leads to an upregulation of p21 ^WAF1/CIP1^, acetylation and induction of p53 and reduction of CHK1 [12, 30].

In all three cell lines, PS led to a strong p21^WAF1/CIP1^ induction. Whereas in NCI-H460 p21^WAF1/CIP1^ was increased even further by PS + erlotinib, in HCC827 the combination antagonized this induction almost entirely. In A549 combination treatment had almost no additional effect (Fig. 3c). In the HCC827 cell line, erlotinib led to a slight increase of p53 after 72 h, whereas with PS or the combination of both drugs p53 levels were modestly decreased. In A549 and NCI-H460, a marked increase of p53 expression was only apparent after 72 h of PS and combination treatment (no additive effect) (Fig. 3c, middle and lower panel).

CHK1 decreased markedly in all three cell lines after 72 h of HDACi treatment. In HCC827 and A549 add-on of erlotinib further enhanced this effect. In line with the AKT immunoblot results, phospho-CHK1 was slightly increased in all three cell lines after erlotinib treatment.

### The panobinostat and erlotinib combination induces an epithelial phenotype in HCC827 and A549 cells

Epithelial to mesenchymal transition (EMT) plays a major role in the metastatic behavior of NSCLC. Also, loss of the epithelial marker E-cadherin is a hallmark in the development of TKI resistance [14]. In HCC827, PS modestly upregulated E-cadherin, indicative of some EMT induction (Fig. 3d, upper panel), as did erlotinib and the combination treatment (72 h). PS alone modestly induced β-catenin expression, whereas the combination with erlotinib had the opposite effect (which appeared to outweigh the effect of HDAC inhibition). A549 did not disclose E-cadherin or β-catenin induction by erlotinib, whereas PS, both alone and in combination with erlotinib, led to an increased expression of both proteins. NCI-H460 showed a different response: PS alone and in combination with erlotinib reduced β-catenin. (No E-cadherin expression was detectable in this cell line, neither before nor after treatment, Fig. 3d, lower panel) [22, 31].

### Erlotinib enhances panobinostat-induced acetylation of histone H3

**Fig. 4:**
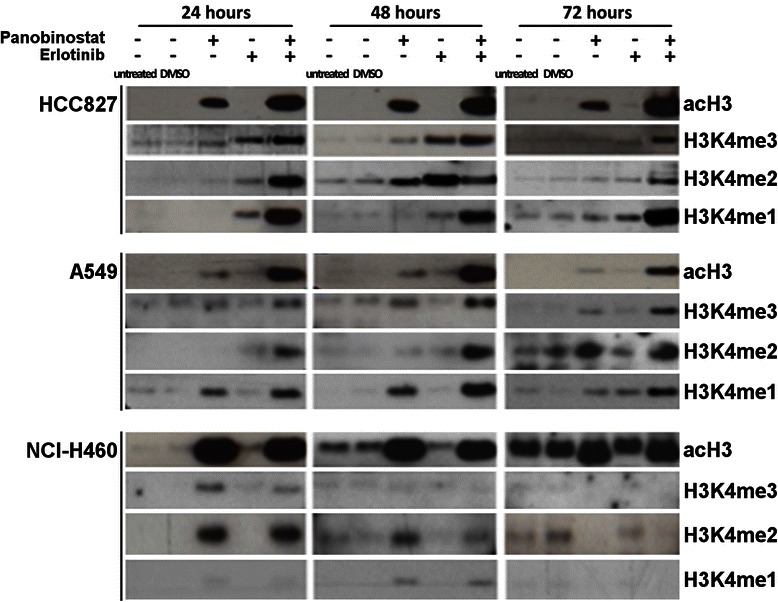
Panobinostat treatment leads to the deposition of activating histone marks which can be enhanced by erlotinib. Non-cytotoxic concentrations of PS induced acetylation of histone H3, which is further increased by combination with erlotinib in both adenocarcinoma cell lines HCC827 and A549. Also, the activating mono-, di- and tri-methylated H3K4 marks were at least additively induced after combination treatment in HCC827 and A549 compared to single agent treatment with PS or erlotinib. In NCI-H460, erlotinib had no additional effect on PS-induced histone acetylation and methylation

As expected, PS robustly induced acetyl-histone H3 (acH3). Erlotinib had no effect on H3 acetylation, but it enhanced the effect of PS on acH3 (in HCC827 and A549). In NCI-H460, it modestly induced acH3 after 24 h, but the combination treatment did not lead to an additive effect compared to PS alone (Fig. 4).

### The combination of panobinostat and erlotinib leads to synergistic induction of activating histone methylation marks in HCC827 and A549

Others [32] as well as ourselves [33], could show that HDAC inhibitors also exert an effect on different histone methylation marks. Therefore we also investigated the histone marks mono-, di-, and trimethylated lysine 4 of histone H3 (H3K4me1/2/3, respectively) by Western blot (Fig. 4). Baseline H3K4 methylation was hardly detectable in all three cell lines. PS induced H3K4me1/2/3 in all three cell lines. Surprisingly, also erlotinib had a positive effect upon methylation of this residue, particularly in HCC827 and, to a lesser degree, in A549 cells. Strikingly, the drug combination exerted a robust synergistic effect on the expression of all three methylation steps of H3K4 in both adenocarcinoma cell lines (Fig. 4).

**Fig. 5:**
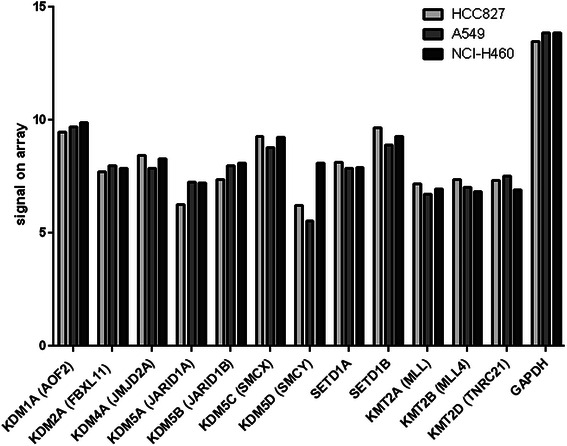
Expression levels of the epigenetically active enzymes of Table 2. mRNA baseline expression levels of the seven H3K4 demethylases and five methyltransferases already depicted in Table 2. Expression levels of the three cell lines were determined by Affymetrix HG-U133A epression arrays by Coldren et al. 2006 and signal intensities as reposited at the GEO database (GSE4342) were plotted. [34]

To investigate if there was a genomic basis for this effect, we also checked for copy number alterations in genes that play important roles in histone methylation by removing or adding methyl groups to H3K4 (i.e. histone demethylases and methyltransferases). We found gains for *KDM4A* and *KDM5A* (HCC827) and of *KDM5B* (NCI-H460). Losses were detected of *KDM1A* and *KDM4A* (A549) and *KDM1A* and *KDM2A* (NCI-H460). No copy number changes could be seen for histone methyltransferases *SETD1A, SETD1B*, *KMT2A/B/C/D* (see Table 2). As we compared these data to already published expression data [34], we could not find a direct correlation between copy numbers and mRNA expression (Fig. 5). These findings suggest that differential methylation of H3K4 is not regulated by copy number alterations of the particular demethylases and methyltransferases. Both PS and erlotinib, alone and in combination, could robustly induce active histone marks independently of genomic amplifications or losses of these enzymes.

**Table 2:** Copy numbers of H3K4-specific demethylases and methyltransferases

gene	KDM1A	KDM2A	KDM4A	KDM5A	KDM5B	KDM5C	KDM5D	SETD1A	SETD1B	KMT2A	KMT2B	KMT2C	KMT2D
HCC827	n	n	3	3	n	n	n	n	n	n	n	n	n
A549	1	n	1	n	n	n	n	n	n	n	n	n	n
NCI-H460	1	1	n	n	3	n	n	n	n	n	n	n	n

Evaluation of copy numbers of seven demethylases and six methyltransferases that specifically target lysine 4 residues of histone H3 (H3K4)

0 = complete loss, 1 = 50 % loss, n = no loss or gain, 3 = 50 % gain and 4 = 100 % gain of genomic material, relative to ploidy (all percentages are approximate values computed with Affymetrix Genotyping Console Software)

## Discussion

The introduction of tyrosine kinase inhibitors antagonizing overactivated or amplified EGFR has strikingly expanded the therapeutic armamentarium in non-small cell lung cancer. However, development of resistance to TKIs is a major clinical problem, since it occurs in almost all patients receiving these drugs for prolonged periods. Therefore strategies to overcome secondary resistance to TKIs have been investigated. These include the usage of epigenetically active agents such as HDACis. In NSCLC, this structurally diverse group of compounds has been tested pre-clinically and in part also already clinically. While single agent HDACi treatment has only modest activity, drug combinations with chemotherapy have been studied, with some trials being positive [9], others being limited by toxicity of the combination [35]. A single phase II study combining erlotinib with the class 1 selective HDACi entinostat has resulted in prolonged progression-free survival in NSCLC patients harboring high E-cadherin levels, irrespective of the *EGFR* genotype [16]. The combination of a pan-HDAC inhibitor like PS with erlotinib has so far been investigated in a phase I trial in aerodigestive tract tumors, which suggested that especially TKI-naïve EGFR-mutated patients might benefit from the combination therapy [13].

In the present study, we attempted to unravel mechanisms of action of the combination treatment of PS with erlotinib in NSCLC cell lines representing different histological subtypes and different genotypes. The combination treatment revealed variable responses: whereas proliferation of the adenocarcinoma cell lines was significantly reduced, the large-cell carcinoma cell line did not respond.

We also hypothesized that the inhibitory effect of erlotinib on EGFR phosphorylation would be enhanced by the HDAC inhibitor, which could indeed be demonstrated for A549 cells and, to a much lesser degree, for HCC827. Cooperative negative effects on downstream signals, e.g. ERK and AKT were also seen only in these two cell lines. Interestingly, in HCC827 erlotinib hardly reduced phospho-EGFR levels and even upregulated phospho-ERK expression. These findings suggest the, already described, partial outgrowth of a TKI-insensitive cell clone. NCI-H460 displayed no change in phospho-AKT expression and phospho-ERK was even upregulated after PS (alone and in combination with erlotinib) treatment, underlining the insensitivity to TKIs as seen in the proliferation experiments.

Similarly, induction of p21^WAF1/CIP1^ and p53 varied broadly, with EGFR wt cell lines being overall more sensitive. Here, especially NCI-H460 showed an additive effect of both drugs on p21^WAF1/CIP1^ expression and p53 was upregulated by PS only. This previously described [28] PS-induced induction of p21^WAF1/CIP1^ and p53 was also present in A549. In HCC827 cells erlotinib counteracted the impact of PS on p21^WAF1/CIP1^ expression; p53 was reduced by PS with no effect of erlotinib. We could also confirm the correlation between HDAC inhibition and p21^WAF1/CIP1^/p53-dependent downregulation of CHK1 [12]. Additionally, we could also detect a modest increase in (and thus inhibition of) phospho-CHK1 after erlotinib treatment. Taken together, this crosstalk between p21^WAF1/CIP1^/p53 upregulation and CHK1 downregulation and inhibition provides a reasonable mechanism for the observed growth inhibition after HDACi treatment.

HDAC inhibitors have been demonstrated to also affect histone methylation, as we could recently show in a model of acute myeloid leukemia [33]. Therefore we further wished to ask whether the antiproliferative effects of PS were associated with deposition of activating histone lysine methylation marks. Indeed, we could demonstrate that global H3K4 methylation levels, which are usually associated with accessible chromatin, were increased by PS in all three cell lines. Unexpectedly, erlotinib as a single agent also induced histone lysine methylation, in HCC827 and A549. Combining both drugs resulted in a synergistic effect on H3K4 methylation (and H3 acetylation), which strongly mirrored the antiproliferative activity of this combination. To the best of our knowledge, a robust effect of erlotinib upon histone methylation has not been previously described. Possibly, induction of histone methyltransferase expression or inhibition of histone demethylase expression may be responsible for this effect.

## Conclusions

Pan-HDAC inhibitors such as PS may provide a viable option to (re) sensitize NSCLC cells, particularly of adenocarcinoma subtype with *EGFR* mutations, to the antiproliferative effects of the TKI erlotinib, warranting further development of this approach within a clinical phase II trial. Our study suggests that the mechanism of action of resensitization involves reactivation of different sets of tumor suppressor genes. We were able to show that this is mediated by inducing an active chromatin configuration via deposition of activating histone marks, such as H3 acetylation and H3K4 methylation. This general strategy may become even more relevant in the future, since resistance to *EGFR* T790M-specific third generation kinase inhibitors, like AZD9291 and Rociletinib (CO-1686), already occur in the clinic which needs to be overcome or might even be prevented [36].
